# The impact of tumour pH on cancer progression: strategies for clinical intervention

**DOI:** 10.37349/etat.2020.00005

**Published:** 2020-04-28

**Authors:** Carol Ward, James Meehan, Mark E Gray, Alan F Murray, David J Argyle, Ian H Kunkler, Simon P Langdon

**Affiliations:** 1 Cancer Research UK Edinburgh Centre and Edinburgh Pathology, Institute of Genetics and Molecular Medicine, University of Edinburgh, Crewe Road South, EH4 2XU Edinburgh, UK; 2 Royal (Dick) School of Veterinary Studies and Roslin Institute, University of Edinburgh, Easter Bush, EH25 9RG Midlothian, UK; 3 School of Engineering, Institute for Integrated Micro and Nano Systems, EH9 3JL Edinburgh, UK; Istituto Nazionale Tumori “Fondazione Pascale” Via Mariano Semmola, Italy

**Keywords:** Tumour pH, acidosis, inhibitor, hypoxia, carbonic anhydrase IX, sodium-hydrogen exchanger 1, monocarboxylate transporter 1, monocarboxylate transporter 4, vacuolar-type H^+^-ATPase proton pump

## Abstract

Dysregulation of cellular pH is frequent in solid tumours and provides potential opportunities for therapeutic intervention. The acidic microenvironment within a tumour can promote migration, invasion and metastasis of cancer cells through a variety of mechanisms. Pathways associated with the control of intracellular pH that are under consideration for intervention include carbonic anhydrase IX, the monocarboxylate transporters (MCT, MCT1 and MCT4), the vacuolar-type H^+^-ATPase proton pump, and the sodium-hydrogen exchanger 1. This review will describe progress in the development of inhibitors to these targets.

## Introduction

**Figure 1.**
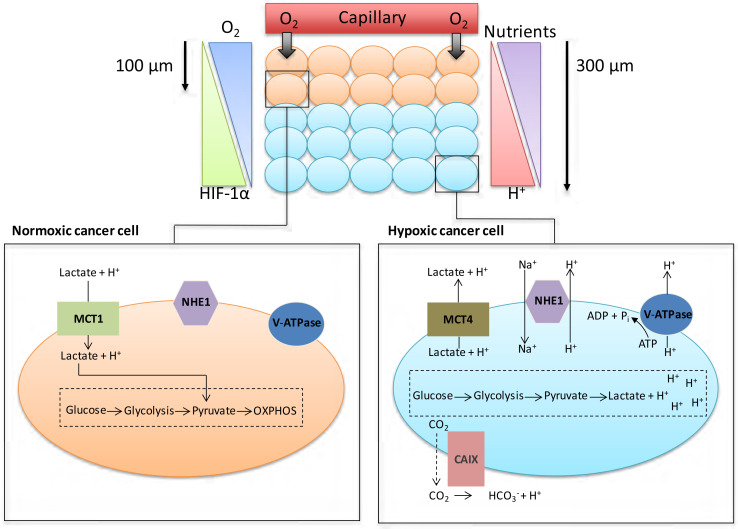
Cancer cell metabolism, HIF and pH. As cancer cells proliferate, some cells are pushed further away from blood vessels, decreasing the levels of O_2_ and nutrients available to these cells. Normoxic cells can produce energy through oxidative phosphorylation (OXPHOS); hypoxic cancer cells are unable to acquire energy through OXPHOS due to low O_2_ levels. Activation of the HIF family of transcription factors is one of the principle oxygen-responsive signalling pathways that allows the adaptation of hypoxic cancer cells to this hostile microenvironment. HIF signalling shifts energy production from OXPHOS in the mitochondria towards glycolysis, allowing hypoxic cancer cells to continue to produce energy despite the low O_2_ levels. The increased dependency on glycolysis in hypoxic cells leads to the production of increased amounts of H^+^ ions, which can lead to changes in the pHi of cancer cells if not dealt with. To help cope with the excess H^+^ ions being produced, cancer cells upregulate/activate a number of pH regulating proteins; these proteins include CAIX, NHE1, MCT4 and V-ATPase

The manifestation of acidic pH in solid tumours is closely connected to the development of areas of low O_2_ concentration (hypoxia). Although angiogenesis occurs in tumours, the resulting vasculature is often malformed and chaotic, causing minimal O_2_ perfusion within cancers and a decreased capacity to deliver nutrients or remove metabolic waste from rapidly proliferating cells. Hypoxic regions develop in tumour regions that are more than 100 μm away from blood vessels (Figure 1). At such locations, there is often a focal necrotic zone, surrounded by a peri-necrotic area, featuring hypoxic and acidic conditions. Cells survive in this environment via adaptive modifications which are largely under the control of the transcription factor hypoxia-inducible factor-1 (HIF-1), a heterodimer formed from α and β subunits. The α subunit is extremely unstable in normoxic O_2_ concentrations, with a short half-life of several minutes because of the O_2_-dependent activation of prolyl hydroxylases; these proteins hydroxylate HIF-1α on two proline residues (402 and 564), directing the interaction of HIF-1α with the Von Hippel-Lindau factor (an E3 ligase), which ubiquitinates the protein and targets it for proteasomal destruction [1]. Conversely, in hypoxia, hydroxylation is inhibited and HIF-1α is stabilised, combines with HIF-1β to form HIF-1, translocates to the nucleus, and activates transcription of target genes. HIF-1α can be stabilised independently of hypoxia by the increased expression of growth factor receptors and the dysregulation of oncogenes, such as the amplification of *c-myc* in cancer cells, and by intracellular lactate accumulation [2, 3].

To maintain intracellular energy levels, cells in hypoxic conditions favour glycolysis, leading to an increased utilisation of glucose and the production of lactate [3]. Many cancer cells use glycolysis for energy production even in O_2_ concentrations that permit oxidative respiration (aerobic glycolysis or the Warburg effect) [4]. HIF-1 activation in hypoxic tumours enhances the expression of various glycolytic enzymes and glucose transporters such as lactate dehydrogenase-α and Glut1, facilitating increased glycolysis [5, 6]. This generates lactate and hydrogen ions that should decrease intracellular pH (pHi), but because modulation of pHi affects crucial cell processes such as adhesion, proliferation, metabolism and apoptosis, it is finely controlled [7]. Cancer cells maintain pHi partially through increased activation and expression of HIF1-dependent genes [6], such as *SLC16A3* [monocarboxylate transporter 4 (MCT4)] which removes lactate along with hydrogen ions from tumour cells, or *CA9* [carbonic anhydrase IX (CAIX)] which facilitates the formation of bicarbonate and hydrogen ions from CO_2_ and H_2_O [8, 9]. However, HIF-2α also plays a role in adaptation of tumour cells to acidosis [10].

Low pH and hypoxia can also activate the sodium-hydrogen exchanger 1 (NHE1), which exchanges intracellular H^+^ ions for extracellular sodium ions [6, 7]. Mechanisms such as these ensure tumour cell pHi remains neutral to slightly alkaline, ranging between 7.1-7.7, as transporters, enzymes and ion pumps maintain the internal cellular environment, while concurrently inducing acidification (acidosis) of the tumour microenvironment (TME) [11, 12]. Normal cellular pHi is generally about 7.2 and frequently lower than cancer cell pHi [13]. pHi has been measured by techniques that include ^31^P-magnetic resonance spectroscopy [14], ^14^C-benzoic acid distribution [15] and use of pH-sensitive fluorescent probes, e.g., BCECFAM probe [14, 15]. Normal cells have an extracellular pH (pHe) value around 7.4 which contrasts with the pHe of cancer cells which can typically vary between 6.7-7.1 [13]. However, this can decrease to as low as 6.0 for cancer cells 300 mm from tumour blood vessels, as lactic acid and protons accumulate extracellularly because the malformed tumour vasculature inhibits their removal, thus reducing the buffering capacity of the extracellular environment [11, 13]. This acidic microenvironment can be toxic to normal cells, but cancer cells adapt and survive [11, 13, 14]. Indeed, studies indicate that while the proliferation rate of normal cells is maximal at pHe 7.3, tumour cell proliferation is highest at pHe 6.8 [14].

## Consequences of acidosis for cancer progression and treatment

### Effect on radiosensitivity and chemosensitivity

The survival strategies adopted by cancer cells in the hypoxic and acidic TME promote radiotherapy and chemotherapy resistance and are clearly connected to the evolution of *in situ* to invasive cancer [11]. For radiotherapy to work effectively, O_2_ must be present at the time of radiation to generate free radicals producing DNA breaks leading to cell death. Under hypoxic conditions, there may be insufficient O_2_ for this radiosensitisation. Hypoxia hinders the homologous recombination (HR), non-homologous end-joining and mismatch repair DNA pathways, as well as inhibiting the G1/S cell cycle checkpoint, allowing DNA errors to accrue and chromosomal instability to increase, while the alkaline pHi of tumour cells hinders mitotic arrest initiated by activated DNA damage checkpoints [13, 16].

Treatment resistance is also linked to acidic pHe, through changes in drug structure and charge (for example, doxorubicin becomes highly charged, inhibiting uptake across cell membranes in low pH) [13, 17], with acidosis also leading to resistance to apoptosis in irradiated cancer cells [18].

### Effect on cancer cell function

Alkaline pHi inhibits apoptosis since activation of caspases requires acidic pHi [19], with the basic pHi also promoting DNA synthesis and cell proliferation [20], thus facilitating increases in cell number and tumour volume. These processes lead to higher rates of mutation in cancer cells [5, 13] and augment cellular survival, causing progression of the disease. A pHi threshold around 7.1–7.2 exists below which growth factors fail to stimulate G1 progression and cell cycle entry [21].

Acidic pHe promotes migration, invasion and metastasis of cancer cells through varied mechanisms; in tumours, the zones exhibiting the most extensive invasion correlate with those displaying the lowest pH, while increased acidity in tumours correlates with poorer prognosis [11, 21–24]. Xenograft studies illustrate a two-fold increase in lung metastases when melanoma cells are exposed to pH 6.8 for 48 h before implantation [22]. Acidic pHe increases the expression of proteinases and the activity of metalloproteases (MMPs) such as MMP-1, 2 and 9, which degrade extracelular matrix (ECM) components, thus aiding the invasion and migration of cancer cells [24–26].

### Effect on the tumor microenvironment

Tumours contain stromal elements including cells of the immune system that should recognise and remove tumour cells and maintain tumour latency [27]. Tumour acidity has been described as a “global protection shield” by which cancer cells neutralise the activity of antitumor immune cells and convert regulatory immune cells to become allies [27].

Cytotoxic T (CytT) cells and natural killer (NK) cells are involved in anti-tumour defences, and large numbers of these cells in a tumour correlate with positive prognosis [28]. Hypoxia and acidosis interfere with immune cell function, hampering this natural defence; acidosis in particular obstructs the activation of both CytT and NK cells, facilitating tumour progression [27, 29–32]. These cells lose their functionality undergoing anergy followed by apoptosis at low pHe [27].

Lactate or acidic pHe inhibits maturation of dendritic cells (DCs) from myeloid precursors, thus hindering antigen presentation [29]. Additionally, acidic pHe can increase expression of cytokines and growth factors [such as IL-8, IL-6 and vascular endothelial growth factor (VEGF)] that support the growth and progression of tumours and angiogenesis, while lactate can activate TGF-β to suppress immune cell function [32–34]. Lactate may also inhibit cytokine release from DCs and CytT cells and interfere with signal transduction [35, 36]. A recent study illustrates that the counteraction of acidic pHe opposes anergic unresponsivenesss in human and murine tumour-infiltrating T lymphocytes [37]. Myeloid-derived suppressor cells (MDSCs) strongly suppress innate and adaptive immunity by inhibiting the anti-tumour functions of T and NK cells, facilitating the maturation of regulatory T cells and hindering the development of DCs. Increased production of lactate by tumour cells promotes MDSC development, suggesting an important role for lactate in the evolution of an immunosuppressive TME [38].

Increased lactate concentrations also stimulate the development of a cancer stem cell phenotype [39]. In glioma, acidic pHe enhanced cell malignancy, promoting expression of stem cell markers such as Oct4 and Nanog, and increasing VEGF levels, via an acidic pH-driven increase in HIF-2α mRNA and protein [40]. Additionally, lactate has been shown to attract human mesenchymal stem cells to tumour cells and heighten stem cell migration [41].

**Figure 2.**
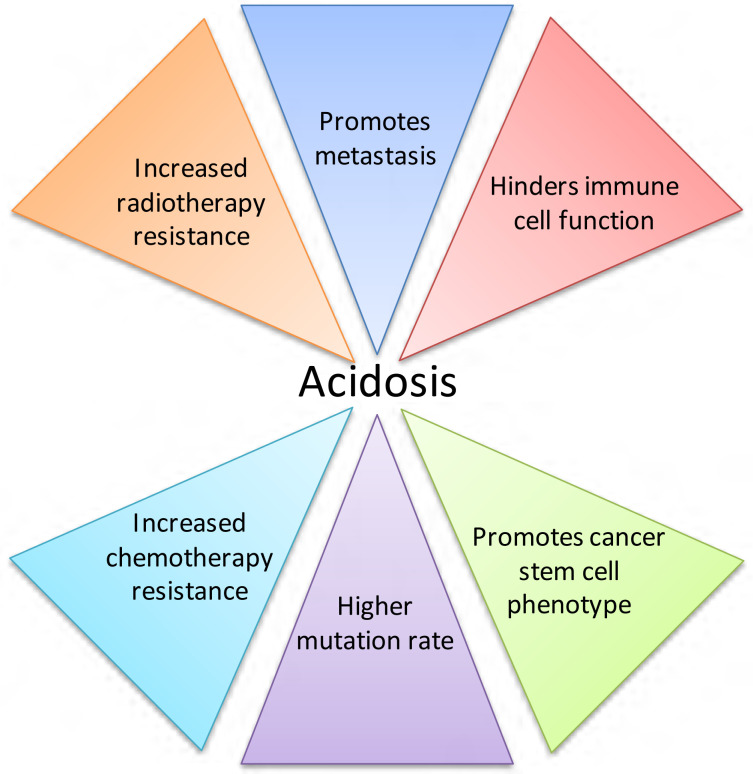
The effects of acidic pHe on tumour development and treatment. Acidosis is a microenvironmental factor that impacts tumour progression greatly, contributing to tumour growth and negatively affecting both radiotherapy and chemotherapy treatment

Activated T cells rely on glycolysis for energy production, but high concentrations of extracellular lactate in the microenvironment prevent lactate export from these cells, since movement is dependent on a lactate gradient between the cytosol and the extracellular space [36]. High extracellular lactate concentrations therefore affect activated T cell function. Consequently, acidic pHe, in concert with increased lactate, may have synergistic effects on the inhibition of immune function in cancer [38]. Lactate also increases cancer cell migration [42], and causes the release of VEGF from endothelial cells, linking lactate with angiogenesis [43]. This is supported by *in vivo* work demonstrating that lactate can cause IL-8-dependent angiogenesis and tumour growth in xenograft models [44]. Lactate accumulation in several tumour types correlates with patient survival, metastatic potential and radioresistance [19, 20, 45]; radioresistance may be caused by the antioxidant effects of lactate [46]. The effects of acidic pHe on tumour development and treatment are summarised in Figure 2. When considered in combination, the consequences of acidosis on tumour growth and progression are substantial.

## Potential therapeutic strategies

**Figure 3.**
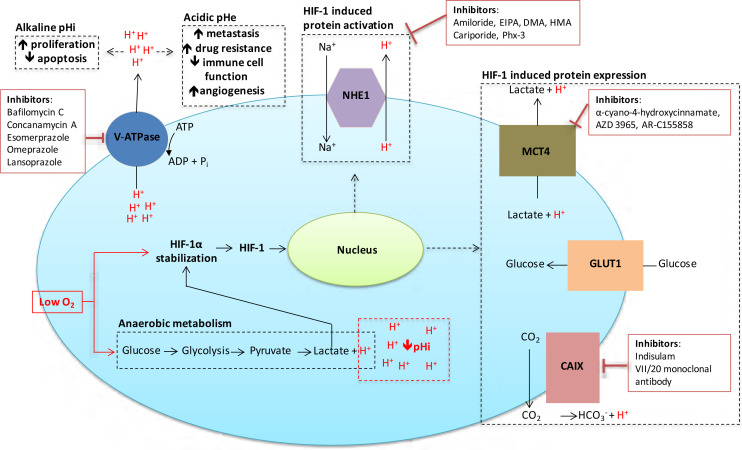
Inhibitors targeted against pH regulatory molecules with the cell. Various drugs targeting the main pH regulating proteins of cancer cells are outlined, along with the effects that alkaline pHi/acidic pHe have on tumour development and treatment

Given the significant roles that acidosis and pH regulation play in the proliferation, survival, invasion and metastasis of cancer cells, this is an area ripe for therapeutic exploitation in the treatment of solid tumours. Here we expand on several possible targets for intervention, in particular CAIX, monocarboxylate transporter 1 (MCT1) and 4, the vacuolar-type H^+^-ATPase proton pump (V-ATPase), and NHE1, all of which have significant functions in the control of cellular pHi [9, 13, 47], as illustrated in Figure 3. Other ion exchangers and transporters are involved in pHi regulation, but their roles in cancer progression are still unclear. A family of proton sensing G protein-coupled receptors [48] that can be activated by acidic extracellular pH may be further targets for tumour therapy.

### NHE1

While there are nine NHE isoforms, this review concentrates on NHE1. Even though this protein is ubiquitously expressed, it has been firmly associated with cell transformation and malignant progression [49], suggesting it has strong potential as a therapeutic target for cancer [50]. This isoform localises to the plasma membrane and is involved in the homeostasis of pHi and cell volume, as well as migration, cell cycle progression, proliferation and cell death [7, 47, 51, 52]. It operates in concert with many signalling pathways and may function as a framework for the interaction of signalling complexes [53]. NHE1 activity is stimulated by hypoxia, decreased pHi, cell transformation, and downstream effectors of epidermal growth factor receptors (EGFR) such as ERK and p90^Rsk^, B-Raf, G protein-coupled receptors, PKC, RhoA, and integrin receptors [6, 54–59]. The NHE1 protein plays a significant role in maintaining pHi by importing extracellular Na^+^ for intracellular H^+^ in a 1:1 ratio [7]. In solid cancers, this leads to acidification of the TME [60], increasing the metastatic potential of cancer cells [61]. For example, higher activity and expression of NHE1 is proposed to increase the potential for invasion and survival of breast and melanoma cancer cells [58, 60–62].

The leading cause of treatment failure in cancer is from metastatic spread of the primary tumour, in which NHE1 is strongly implicated through increased metalloproteinase activity leading to digestion of extracellular matrix components [63]. NHE1 interacts with CD44, a transmembrane glycoprotein that functions in cell adhesion, migration, invasion and survival [64]. This interaction activates hyaluronidase-2 and cathepsin B allowing invasion of breast tumour cells [24]. A positive correlation between CD44 and NHE1 has been described, where knockdown of CD44 caused a decrease in NHE1 mRNA and protein expression which was associated with the suppression of migration and invasion of breast cancer cells; this was reversed by increasing NHE1 expression in cells where CD44 expression was impaired [65]. NHE-1 is expressed in lamellipodia and invadopodia, where it functions in cell motility and directional migration [66, 67]. CD44 is also located in invadopodia, where it stimulates NHE1 activity and invasion via the RhoA effector ROCK1 [23, 58].

NHE1 secures the cytoskeleton to the cell membrane by attaching to ezrin/radixin/moesin proteins and talin, which connect to actin, thus in part, explaining the involvement of NHE1 in cell motility [62, 68]. It partially controls the pH-dependent activation/integration of cytoskeletal components involved in the regulation of actin polymerisation, such as ADF/cofilin, talin and gelsolin, allowing refashioning of cell-substrate adhesion complexes required for migration [62, 69, 70]. As part of these focal contacts, NHE1 may co-operate with integrins and other cell signalling complexes [7]. Increased expression of the ErbB2 tyrosine kinase is associated with many types of cancer. In its N-terminally truncated isoform (ErbB2t), it becomes constitutively active leading to poorer patient prognosis [71]. This is associated with an NHE1-dependent mechanism, since ErbB2t expression is related to acidification of the pericellular space which is sensitive to NHE1 inhibition [72]. NHE1 regulates motility of breast cancer cells expressing ErbB2t and stimulates EMT development [73].

Because acidic pHi is optimal for activation of apoptotic proteins such as caspases and endonucleases, proteins that maintain alkaline pHi can reduce activation of apoptotic pathways and protect against programmed cell death [17]. Increased expression of NHE1 leads to decreased responsiveness of several known inducers of apoptosis in tumour cells, while reduced expression sensitises cells to apoptosis [74, 75]. NHE1 is a caspase-3 substrate, therefore cleavage of NHE1 may be part of the apoptotic process [76]. The PKB/Akt survival pathway is stimulated by association of NHE1 and ezrin, and in turn, PKB/Akt can stimulate NHE1 activity [77, 78], suggesting that NHE1 may increase cancer cell survival per se, regardless of its role in pHi maintenance. However, NHE1 inhibition can block Akt phosphorylation, suggesting that Akt activity may be dependent on alkaline pHi [79]. Inhibition of NHE1 sensitises cancer cells to etoposide, paclitaxel and daunorubicin [74, 80, 81]. However, paclitaxel itself can inhibit NHE1, and this may be causal in the induction of apoptosis induced by this and other cancer therapeutics, or the inhibition may be due to apoptotic cleavage of NHE1 as previously mentioned [75, 79–81]. Additionally, NHE1 inhibition or depletion reversed cisplatin resistance in breast cancer cells, doxorubicin resistance in human colon cancer cells [69, 80, 82], and caused growth arrest, lowering of pHi and increased responsiveness to cell death inducers in cholangiocarcinoma cells [74, 83]. In leukaemia cells, NHE1 inhibition reduced proliferation and VEGF synthesis [84], suggesting that NHE1 may play a role in angiogenesis. In support of this, a recent study demonstrated that NHE1 suppression using siRNA could inhibit HIF-1α-induced angiogenesis in a human umbilical vein endothelial cells (HUVEC) model [85]. Peroxisome proliferator-activator receptor gamma ligands reduce cancer cell division by repressing NHE1 expression [80], while xenograft models demonstrate that deficient NHE1 activity or NHE1 deletion inhibits tumour growth [86, 87]. However, earlier work using the NHE1 inhibitor amiloride demonstrated a delay or inhibition of apoptosis induced by radiation in HL60 cells [88], although this is not a solid tumour model. Taken together, these studies suggest targeting NHE1 activity may induce growth arrest in cancer cells and increase sensitivity to anticancer treatment.

Some specific inhibitors of NHE1 (see Table 1), such as cariporide (HOE642) and eniporide have concluded phase II/phase III clinical trials [89, 90] in a cardiological or ischaemic-reperfusion injury setting. An early study using cariporide suggested that NHE1 inhibitors could have therapeutic benefit in reperfusion injury, leading to further clinical trials [91–93]. Although cariporide reduced myocardial infarcts, it was linked to increased cerebrovascular events, causing the trials to be stopped [90, 92]. This may have been due to the use of higher cumulating doses of the drug [94], but these trials allowed a tolerable dosing schedule to be developed. Cariporide has never been used clinically in cancer patients, but if the undesirable side effects could be dissassociated from therapeutic gain by minimising the dose, it could form part of a treatment schedule. Its use in cancer patients is supported by early preclinical studies which demonstrated activity in leukemia and cholangiocarcinoma cells [12, 95–97]. Recently, preclinical activity of cariporide has been demonstrated across a broad range of cancer types [98–103]. The drug has been shown to inhibit invasion of head and neck squamous cancer [98] and EGFR driven invasion of pancreatric cancer [99]. Cariporide can sensitise breast cancer cells to doxorubicin both *in vitro* and *in vivo* [100] and can inhibit accelerated net acid extrusion in human colon cancer biopsies [101]. The agent has also been shown to modulate pHi of human glioblastoma xenografts implanted into mice brains [102]. Combined with the MCT1 inhibitor, AR-C155858, it has an additive inhibitory effect on leukemia cells [103].

**Table 1.** Therapeutic targets and inhibitors

Target/Inhibitor	Examples of preclinical sensitivity	References
NHE1		
Amiloride	Mammary, prostate, gastric, hepatoma	[104]
Ethylisopropylamiloride (EIPA)	Bladder, breast cancer	[109]
Dimethylamiloride (DMA)	Bladder, breast cancer	[109]
Hexamethyleneamiloride (HMA)	Leukemia	[110]
Cariporide	Leukemia, cholangiocarcinoma	[12, 96–103]
Phx-3	Gastric cancer	[111, 12]
MCT1/MCT4		
α-cyano-4-hydroxycinnamate	Lung cancer	[116]
AZD 3965	Small cell lung cancer	[131]
AR-C155858	Rat transformed fibroblasts	[121]
V-ATPases		
Balifomycin C	Pancreatic cancer, colon cancer	[157, 159]
Concanamycin A	Submandibular cancer	[162]
Esomerprazole	Breast cancer, esophageal cancer	[171, 172]
Omeprazole	Melanoma	[168, 169]
Lansoprazole	Pancreatic cancer, breast cancer	[166, 170]
CAIX		
Indisulam	Lung cancer	[208]
VII/20 Monoclonal antibody	Colon cancer	[198]

Amiloride, another NHE1 inhibitor, is well tolerated as a diuretic [104] and direct antitumoural, antimetastatic and antiangiogenic effects have been reported [105–107]. Analogues of amiloride that are more specific NHE1 inhibitors such as ethylisopropylamiloride (EIPA), dimethylamiloride (DMA), and hexamethyleneamiloride (HMA) also have anticancer potential [108–110]. A phenoxazine structure, Phx-3, has also shown interesting preclinical activity [111]. Improved specificity and/or delivery of NHE1 inhibitors may allow further exploitation of this anti-cancer target.

### Monocarboxylate transporters (MCTs) 1, 4 and associated molecules

MCTs 1 and 4 enable movement of lactate and protons across cell membranes and therefore also function in pHi regulation [112]. These isoforms are ubiquitously expressed, with MCT4 expression strongly enhanced in glycolytic tissues [112]. MCTs levels are increased in many cancers such as breast, colorectal, ovarian, and prostate, and are associated with disease progression and poor prognosis [113–115].

The activation of HIF-1 suppresses the expression of MCT1 while increasing that of MCT4; this maintains an economical use of glucose in the tumour [8] since the lactate produced by hypoxic cells is exported along with hydrogen ions from the cell by MCT4, and can be transported into cells in more oxygenated areas by MCT1 [8, 116]. Where O_2_ concentrations are sufficient for oxidative phosphorylation, cells can use lactate in the tricarboxylic acid cycle. This allows for the development of a symbiotic association between cells in solid tumours [116]. Stromal cells, such as tumour-associated fibroblasts and endothelial cells, demonstrate increased MCT1 expression and can therefore ulitise lactate [44, 117]. Blocking this symbiotic mechanism should prevent lactate use by normoxic tumour cells, increasing dependency on glucose, and decreasing glucose availability for hypoxic tumour cells. Although inhibition of MCT4 alone could be sufficient to block this symbiotic relationship one study using a mouse model has shown that MCT1 inhibition can also effectively target cells in hypoxic areas of lung and colorectal tumours and increased the efficacy of radiation treatment [116]. Therefore, obstructing the activity of MCT1 or MCT4 may be a useful therapeutic target, both in terms of disrupting the efficient usage of glucose and lactate, and also for intervention in pH regulation.

Both MCT1 and 4 are associated with cancer cell invasion and drug resistance [118, 119]. By obstructing intracellular acidification, MCTs also inhibit the induction of apoptosis [13], while lactate import into vascular endothelial cells via MCT1 stimulates angiogenesis [120]. Therefore, MCT inhibitors could decrease tumour growth by reducing neovascularisation and cell survival, while limiting invasion and counteracting drug resistance. Preclinical studies indicate that reducing MCT1 activity diminishes the growth of a diverse range of tumours such as lymphoma, glioma, and colon cancer [116, 121–123]. The MCT1 inhibitor α-cyano-4-hydroxycinnamate sensitised colon cancer cells to cisplatin by a mechanism involving the down regulation of MDRI and MRP1 genes, which are involved in multidrug resistance (MDR) [122]. This may be via an effect on pHi since prior reports have illustrated that pHi has a pivotal involvement in MDR development [124] through regulating MDR gene expression [125]. Disturbing the function of MCTs causes intracellular lactic acid to accrue and this in turn reduces glucose transport, ATP production and pHi, hindering growth of human cancer cells [126]. *In vivo* preclinical studies established that MCT1 inhibitors delayed the onset of tumour growth; growth was inhibited in mice co-treated with both MCT1 inhibitors and metformin, with many mice not developing tumours at all [126]. In another report, inhibition of MCTs increased cell death in human colon cancer cells; this was associated with decreased lactate secretion and a fall in pHi [127]. Reduction in lactate secretion has been linked to increased radiosensitivity in small cell lung cancer xenografts treated with an MCT1 inhibitor [128]. Therefore, MCT inhibitors are appealing therapeutic agents (see Table 1). Preclinical studies have shown that MCT inhibitors exhibit efficacy with some of these drugs now in clinical trials [129, 130]. For example, AZD3965 is undergoing phase I clinical trials in prostate and gastric cancer and diffuse large B cell lymphoma (http://www.clinicaltrials.gov/show/NCT01791595) [131].

Plasma membrane expression of MCT1 and MCT4 requires co-expression of the ancillary molecule CD147, which appears to be HIF-1α-dependent in lung, breast and liver cancer [132, 133]. CD147 is an obligatory assembly factor for MCTs [133]. In breast and pancreatic cancer cells, CD147 silencing strongly reduces glycolysis and lactate secretion [134, 135], and may prevent transformation and tumour development via MCT loss of function [121]. Glucose deficiency can induce ROS-dependent stabilisation of both MCT1 and CD147 [135]. These MCT1-CD147 complexes facilitate tumour cell migration towards glucose, and thus migration away from the low glucose TME [135]. Such complexes are often found colocalised with β1 integrin in the lamellipodia of migrating cells [8] and can act as a framework for other proteins functioning in migration [136]. Consequently, expression of CD147 is increased in metastatic breast cancer cells and other aggressive tumours [112–115, 137], while studies show that decreasing CD147 expression inhibits migration of invasive cancer cells [138]. CD147 also stimulates production of MMPs, which facilitate migration [139]. In A549 cells, increased CD147 expression was found to reduce apoptosis, while also increasing the invasive potential of these cells in hypoxic conditions [130]. Several reports further suggest a role for CD147 in radioresistance. For example, high expression levels of CD147 in cervical squamous cell carcinoma patients were found to be indicative of radiation resistance and poor prognosis [140]. *In vitro* and *in vivo* studies showed that CD147 knockdown significantly enhanced the radiosensitivity of hepatocellular carcinoma cells and decreased radiation induced migration and invasion [141] through a mechanism that inhibited β1 integrin signalling. Therefore, CD147 is another focus for cancer treatment that could be used to disrupt tumour pH homeostasis and glucose/lactate symbiosis.

### H^+^-associated ATPases

V-ATPases are ATP-dependent multi-subunit enzymes that translocate protons across membranes, playing a significant role in the maintenance of pHi homeostasis, and are involved in cell survival [142]. They comprise of a V_0_ region, composed of five different subunits, that is involved in proton transport and a V_1_ sector, involving eight different subunits, which is concerned with ATP hydrolysis [142]. They are primarily situated in the membranes of endosomes and lysosomes and the plasma membrane of some tumour cells [142–144]. The role of V-ATPases in the control of vacuolar pH influences the pharmacokinetics of chemotherapeutic drugs and may be involved in multidrug resistance mechanisms and the metastatic potential of tumour cells [143–146]. Irradiated cancer cells can develop autophagic vesicles which are acidified by V-ATPase; this acidification is restricted by V-ATPase inhibitors and can sensitise these cells to radiation [147]. Therefore, the inhibition of this enzyme is a plausible intervention strategy in cancer treatment (see Table 1).

Increased expression of V-ATPases occurs in various tumours such as hepatocellular carcinoma, oral squamous cell cancer, pancreatic cancer and melanoma [148, 149]. Studies illustrate that V-ATPase inhibitors or siRNA knockdown of V-ATPases can restrain tumour growth and metastatic potential, increase cell death and resensitise tumour cells to chemotherapeutics [146, 150–153]. Inhibition of V-ATPase induces loss of cell adhesion and cell death in colon carcinoma cells and antiproliferative activity in other human carcinoma cell lines (such as ovarian, breast, lung, prostate, hepatocellular, melanoma and renal), and has been shown to reverse trastuzumab resistance in breast cancer cells by interference with HER2 signalling pathway and cellular location [150, 153–155]. *In vitro* models demonstrate that V-ATPase inhibitors or siRNA knockdown hinder the invasion of breast cancer cells through matrigel [143, 144]. Rab27B-dependent invasive growth and metastasis of breast cancer is reliant on V-ATPase activity [156], with the V-ATPase V1E protein expressed in the majority of breast cancer patients with poor prognosis [156]. Classic V-ATPase inhibitors such as bafilomycin A1 and concanamycin A restrain tumour growth *in vitro* and *in vivo* through decreasing the pHi of cancer cells, and have also been shown to induce apoptosis [157–159]. They inhibit the interaction of HIF-1α with VHL, increasing HIF-1α expression in cancer cells, but without enhancing transcription of HIF-1 target genes. Rather, this HIF-1α interferes with binding of C-MYC to the CIP1 promoter, causing increased expression of p21^*CIP1*^ and inhibiting cell cycle progression [160].

Bafilomycin A1 and concanamycin A are unsuitable for clinical use because of toxicity. However, recent studies have determined thata subunit of the V_0_ domain determines the intracellular location of V-ATPase. There are 4 different isoforms of this subunit, and of these the a3 and a4 isoforms are involved in targetting V-ATPase to the plasma membrane [143, 161]. Higher plasma membrane expression of V-ATPase is associated with metastatic cancer cells and knockdown of these subunits inhibits the invasion of breast cancer cells and bone metastasis of mouse melanoma cells [143, 148, 161]. Concanamycin A has been shown to induce apoptosis in human submandibular gland ductal cancer cells [162]. Therefore, future strategies may involve specific targeting of the a3 or a4 subunits, thus reducing overall toxicity by limiting the inhibition of V-ATPase expressed intracellularly.

Another approach involves the use of proton pump inhibitors (PPIs), such as esomerprozole and omeprazole that target H^+^/K^+^ ATPases, since they also inhibit V-ATPase activity; these drugs have long been used efficaciously as therapy for acid-related diseases such as peptic ulcers with minimal side effects [163, 164]. These compounds have anti-inflammatory, immunomodulatory and anti-metastatic properties; they can act as anti-oxidants, interact with neutrophils, monocytes, epithelial and endothelial cells, and inhibit the binding of cancer cell integrins [165]. For example, the PPI lansoprazole prevented cancer cell binding to ECM components [166] and the formation of lung metastases by murine colon cancer cells [165].

PPIs sensitise cancer cells to cytotoxics such as cisplatin, 5-fluorouracil and vinblastine [145], and because PPIs are weak bases, the active protonated form of the drug accrues in the acidic TME [167]. These compounds induced cell death in human B-cell xenografts via a ROS-dependent, caspase-independent mechanism [152], and have shown antitumour activity against melanoma *in vitro* and *in vivo* via a caspase and pH-dependent mechanism [168]. Pretreatment with omeprazole increased cisplatin effectiveness in a murine melanoma model [145] and inhibited invasion and metastasis of breast cancer cells by downregulating the expression of MMP-9 and the chemokine receptor 4 (CXCR4) [169]. Another compound, lansoprazole, induced apoptosis in breast cancer cells and xenografts, inhibiting tumour growth *in vivo*, with no evidence of systemic toxicity [170], while an additional pre-clinical study has shown that the PPI esomeprazole can increase the effectivess of doxorubicin in triple negative breast cancer cells [171]. The compound has also demonstrated acivity against esophageal cancer cell lines [172]. Furthermore, esomeprazole enhanced the activity of tumour-infiltrating T lymphocytes in a murine melanoma model, delaying cancer progression and increasing the efficacy of anticancer vaccines [37], suggesting that PPIs may be useful in reversing the immune evasion mechanisms dependent on acidic pHe and also increasing the effectiveness of anti-cancer immunotherapy treatments.

PPI use may decrease the development of oesophageal adenocarcinoma and hepatoblastoma [173, 174]. Over a 5 year period, one report confirmed that PPIs such as omeprazole, esomeprozole, rabeprazole, pantoprazole or lansoprazole significantly decreased both the growth and progression of Barrett’s oesophagus [174]. A multi-centre phase II clinical trial examining the ability of esomeprazole to sensitise osteosarcoma to neo-adjuvant chemotherapy found an increased response, particularly in chondroblastic osteosarcoma, with no serious side effects reported by patients [175]. In a phase I/II clinical study in veterinary canine and feline patients with spontaneously occurring tumours showing chemotherapy resistance, 67.6% of treated animals demonstrated partial or complete clinical response with a high dosage PPI/chemotherapy treatment [176]. Combination treatments of high dose esomeprazole with cisplatin or docetaxel in metastatic breast cancer in a phase II clinical study (ClinicalTrials.gov Identifier: NCT01069081) reported enhanced responses to chemotherapy with no increase in toxicity [177]. This study demonstrated an increased objective response rate and time to progression with combination treatment and was particularly effective in triple negative breast cancer patients.

### Carbonic anhydrase IX

Carbonic anhydrases (CAs) catalyse the reversible conversion of CO_2_ and H_2_O to HCO_3_^−^ and H^+^. These proteins are ubiquitously expressed and are mainly involved in pHi regulation; this review considers the role of CAIX, since increased expression of this protein is associated with poor prognosis in several cancer types [9]. The extracellular HCO_3_^−^ generated by CAIX is transferred into the cytosol where it limits acidification, while protons remain at the cell surface lowering the pHe [178]. CAIX is a dimeric protein with an N-terminal proteoglycan domain, a CA catalytic domain, and a transmembrane region with a small cytoplasmic tail at the C-terminus [179].

Activation of HIF-1 increases the expression of CAIX, particularly in solid, hypoxic tumours [47]. Expression is limited in normal tissues [9], and knockout of the mouse CAIX homolog causes little abnormality other than gastric hyperplasia [180], suggesting that toxicity in normal tissue by strategies that target this molecule should be limited. CAIX enhances tumour cell survival and growth by regulating pHi homeostasis [14, 47], but it may also influence survival signalling pathways, since limiting CAIX expression significantly affected cell growth and survival independently of O_2_ concentration in breast tumour models [181]. Further, EGF can induce phosphorylation of the intracellular domain of CAIX, causing an interaction with PI3K and Akt activation in some cancer cells such as clear cell renal cancer [182]. Disregulated PI3K activation in tumours can enhance CAIX expression via a mechanism dependent on HIF-1α translation by mTOR [183]. Recent *in vivo* research demonstrated that CAIX knockdown reduced xenograft tumour volume in breast and colon cancer models, and when used in concert with antiangiogenic therapy, reduced growth additively [14, 184, 185]. Conversely, CAIX overexpression in a colon cancer spheroid and xenograft model showed enhanced rates of growth and increased expression of the proliferation marker Ki-67 [185].

CAIX is strongly associated with the cell membrane and cell adhesion, via an interaction with β-catenin that may inhibit E-cadherin-dependent cell-cell adhesion [179, 186]; it functions in both cell migration and invasion. At the C-terminal intracellular tail, Thr-443 can be phosphorylated by PKA in hypoxic conditions, allowing migration to occur [187] via modification of transcriptional activity and proteins involved with EMT and cytoskeletal composition [188], and also in alterations in Rho/ROCK signalling that can stimulate paxillin, and focal adhesion turnover [188, 189]. Recently, CAIX was found to be involved in cancer cell migration and MMP14-mediated invasion by supplying the protons needed for the catalytic activity of MMP14 [190].

The invasive potential of human ductal breast cancer spheroids, along with renal and ovarian cancer cells, was decreased when CAIX expression was reduced or activity was inhibited using novel sulfamate inhibitors of CAIX [191–193]. These inhibitors reduced proliferation, enhanced the rates of apoptosis and inhibited metastasis in breast cancer xenografts models, while also exhibiting the potential to reverse the invasion of human breast tumour biopsy tissue [185, 192, 194, 195]. Basal like breast cancers frequently express CAIX and have been associated with high levels of brain, lung and bone metastases [194], leading to suggestions that targeting of CAIX may be a possible approach to combat metastatic disease [194, 195]. Of note, *in vivo* studies conducted with these inhibitors did not appear to induce toxic responses.

Several novel inhibitors and antibodies targeting CAIX have been assessed as potential anti-cancer treatments [195–198] (see Table 1). SLC-0111/WBI5111 inhibits both tumour growth, metastasis and stem cell numbers either alone or in combination with other anti-cancer therapeutics and is undergoing clinical trials (NTC02215850) [199, 200]. One monoclonal antibody has proved efficacious in colorectal cancer xenografts [198]. Several clinical trials have been undertaken to assess the effectiveness of the antibody girentuximab (cG250), the most clinically advanced monoclonal, in renal cancer (NCT00087022, NCT02002312). Phase I and II trials established safety, tolerability and a positive effect on the disease either alone or in combination with IL-2 treatment [201–203]. In a phase III trial NCT00087022, cG250 showed no effects on disease free survival on non-metastatic renal cell carcinoma patients; however, this study did not stratify patients by CAIX expression. Subsequent analysis demonstrated significant improvement in patients with high CAIX expression, suggesting patient selection is necessary for CAIX-based therapies [204]. CAIX mAbs have been developed that allow delivery of radioisotopes and cytotoxic drugs to cancer cells [205, 206]. A phase II trial of ^177^Lu-girentuximab in metastatic clear cell renal carcinoma attained stable disease in 9 of 14 patients [207]. Another inhibitor indisulam, which demonstrated promising preclinical activity [208], has undergone phase II clinical trials for stage IV melanoma, renal clear cell carcinoma and metastatic breast cancer [209], although in advanced NSCLC patients there was little response when it was used as second-line therapy [210].

Novel CAIX inhibitors have been found to increase sensitivity to radiation [192, 211], suggesting possible combination treatment strategies. Knockdown of CAIX alone or both CAIX and CAXII enhanced the response of tumour cells to radiation, implying that CAIX may protect cells by maintaining alkaline pHi and preventing cell death [212]. Another study demonstrated the sensitisation of renal cell carcinoma to radiation using CAIX inhibitors, where increased levels of apoptosis were observed [213]. Similar results were found in a breast cancer model, where proteomics suggested that CAIX inhibitors in concert with radiation reduced the expression of anti-apoptotic proteins, while increasing that of pro-apoptotic proteins [192]. These results are supported by other studies in xenograft models of colon cancer [211, 214]. CAIX may also affect cancer cell responses to radiation via mechanism that are not pH-dependent, such as interactions with EGFR, PI3K/AKT and NF-kB signalling pathways [215]. Further new inhibitors are in pre-clinical assessment [192, 195, 196], and a phase I clinical trial using a novel dual CAIX inhibitor/radiosensitiser DTP348 has been recently announced (NCT02216669).

## Novel pH targets

### Proton sensing G protein-coupled receptors

A family of five G-protein coupled receptors (GPCRs) involved in proton sensing influence many tumour cell activities such as proliferation, apoptosis, metastasis and angiogenesis; these proteins may therefore offer further targets for anticancer treatments [216–226]. These GPCRs [GPR4, GPR65 (TDAG8), GPR68 (OGR1), GPR81 (HCAR1) and GPR132 (G2A)] are activated by acidic pHe via protonation of histidine residues [216, 217, 225–230]. They are not directly involved in proton translocation, but link to various signalling pathways, such as cAMP, phospholipase C/Ca^2+^, and Rho [216, 217, 225, 226, 231].

Enhanced activation or expression of proton transporters is linked to increased tumour progression, but the stimulation or overexpression of some GPCRs is associated with anti-tumour effects. For example, activation of GPR4 in acidic conditions reduces migration, invasion and the metastatic potential of tumours, while repressing vascular growth [217, 219]; this GPCR activates the G_s_, G_12/13_ and G_q_ G-protein pathways [216, 220, 221, 232]. In melanoma and prostate cancer cells, ectopic expression of GPR4 activated by acidic pH decreases migration and melanoma lung metastases, but it does not significantly decrease growth of the primary tumour [220, 232]. However, GPR4 deficiency in murine models reduces tumour allograft growth by interfering with angiogenesis since GPR4 is expressed on endothelial cells [217, 223]; however, overexpression of GPR4 can transform fibroblasts [233]. This suggests that GPR4 may have both anti and pro-tumour effects dependant on cell type, which may limit the clinical application of compounds that increase or limit GPR4 activation, until further research clarifies the possible mechanisms involved.

GPR68 overexpression impeded migration of both breast and prostate tumour cells and metastasis of prostate cancer cells; although this effect may be independent of pH [219, 234]. It also inhibited ovarian cancer cell migration and augmented adhesion to several ECM proteins [222]. However, GPR68 expression appears to be lower in metastic tumours when compared with the primary growth [218]. GPR68 deficiency significantly reduced tumourigenesis of melanoma cells in a mouse model [235]. Recent research suggests that this GPCR can partially control the interactions between cancer-associated fibroblasts (CAFs) and pancreatic ductal adenocarcinoma (PDAC) cells. TNF-α, secreted by PDAC cells, enhances expression of GPR68 on CAFs via a mechanism involving cAMP/PKA/CREB signaling; activated GPR68 causes secretion of IL-6 from CAFs and this in turn increases the proliferation of PDAC cells [236]. Another study has shown that the transformation of mesenchymal stem cells to CAFs in acidic pHe is dependent on GPR68 [237]. Hypoxia may also control the expression of GPR68 in some cells such as macrophages since HIF-1α binds to the promoter of this gene and its expression is further enhanced by acidic pHe [238].

GPR65 is usually confined to lymphoid cells, but it behaves as an oncogene in lymphoid and other cancers [239]. GPR65 is activated at pH levels lower than 7.4, but activation is maximal between pH 6.2 to 6.8, which is within levels measured in the TME [226, 228]; the ability of this molecule to act in a tumourigenic manner is dependent on its pH sensor activities [240]. High expression of *GPR65* mRNA has been detected in kidney, ovary, colon and breast tumours amongst others [233]. Increased levels of GPR65 were associated with the growth of lung cancer cells and the transformation of mammary epithelial cells [233, 234]. In a murine model, overexpression of GPR65 increased tumour growth via a mechanism that may involve adaptation to acidic conditions through activation of PKA and ERK; knockdown of this receptor inhibited survival of lung cancer cells in an acidic environment [240]. Enhanced cAMP (which activates PKA) production in response to acidic pHe by this sensor has also been reported [228, 229]. Acidic activation of GPR65 upregulates expression of Bcl-2 via MEK/ERK signaling and can inhibit apoptosis of glutamine starved cells [241]. Lymphoma cells and lymphocytes express high levels of GPR65 [242]. In a mouse model, GPR65 knock-out does not produce abnormalities, suggesting this receptor might be a potential therapeutic target [243].

GPR81, a G protein-coupled lactate receptor, is expressed in some tumour cells; stimulation of this receptor helps modulate the effects of lactate by modifying MCT and CD147 expression, aiding tumour growth and metastasis [227]. The GPR81/lactate pathway also suppresses immune surveillance; GPR81 activation causes inhibition of PKA via a decrease in intracellular cAMP levels, aiding activation of the PDL1/PD-1 immune checkpoint and diminished T cell function [244].

GPR132 was initially discovered to inhibit tumour development by hindering cell cycle progression and mitosis; but high levels of expression in fibroblasts caused transformation in one study [245, 246]. Recently, GPR132 has been shown to be involved as a macrophage lactate sensor in the acidic TME during metastasis of breast cancer. Lactate stimulates GPR132 on tumour macrophages, promoting the development of an M2 phenotype that enables migration and invasion of tumour cells [247]. Clinical data show a positive correlation in breast cancer between GPR132, M2 macrophages, metastasis and poor patient prognosis, while GPR132 deletion in a mouse model of breast cancer inhibits metastasis to the lung [248]. Interestingly, expression of GPR132 is almost non-existent in breast cancer cells, suggesting that anti-metastatic effects occur via stromal elements in the tumour [248]. GPR132 expression appears to be suppressed by PPARg activators such as the thiazolidinediones, which may suggest these as possible anti-metastic treatments in breast cancer [248].

Further research is required to fully understand the role of pH-sensing GPCRs in particular cancer types, but small molecule modulators are under development and evaluation; this may help to define their functions. GPR4, GPR68 and GPR65 antagonists are now available [249, 250].

### Histone acetylation

Histone acetylation causes chromatin to adopt an open state, increasing the accessibility of transcription factors. Histone acetylation may also be involved in regulation of pHi [251]. A fall in pHi was associated with lower levels of histone acetylation, which was dependent on the actions of histone deacetylases (HDACs), which are overexpressed in many cancers. Activation of HDACs caused an increase in acetate anions which were transported extracellularly along with protons via MCTs. Increased pHi caused a reversal of transportation. This suggests that histone acetylation can function to regulate pHi and that inhibition of HDACs may form another strategy to compromise tumour cell pH regulation [251]. Interestingly, decreased histone acetylation has been associated with more aggressive cancers and poorer prognosis in breast, prostate and pancreatic cancers [252–255], with overexpression of particular HDACs correlating with disease-free survival, overall survival and poor patient prognosis in several solid tumour types [256], while HDAC inhibitors (HDACi) are known to lower pHi in colon carcinoma cells and xenografts [255]. Numerous preclinical studies have shown increased radiation sensitivity in reponse to co-treatment with HDACi. For example, studies in prostate cancer and NSCLC cell lines showed that HDACi could increase radiosensitivity in a time dependent manner in both hypoxic and normoxic conditions, increasing apoptosis and DNA damage [257, 258]. HR-related gene expression can be reduced by these inhibitors, which may partially explain their ability to radiosensitise cells [259].

There are currently 14 HDACi involved in over 130 clinical trials as monotherapy or in combination with radiation/chemotherapy in a variety of tumours [256]; three are now FDA-approved for use in cancer treatment [260, 261]. Clinical trials in prostate, kidney and bladder cancer were recently reviewed [262]. However, little or no response has been seen in most solid tumours to HDACi alone; most trials now use HDACis in combination with other therapies [261, 263]. HDACi such as vorinostat and valproic acid have been assessed in combination with radiotherapy [259]; a phase I trial (NCT00670553) on prostate cancer has concluded. Vorinostat sensitises glioblastoma cells to radiation, and clinical trials (NCT00731731) of this HDACi in combination with temozolomide and radiotherapy have been undertaken [264]. Phase II studies have also been conducted in combination treatment with paclitaxel (in advanced gastric cancer) [265], and tamoxifen (in hormone therapy resistant breast cancer), where a clinical benefit rate of 40% was achieved [266].

## Innovative technology exploiting the tumour microenvironment

Although the lowered pH in the TME can be linked to cancer progression and poor prognosis, it can also be exploited for cancer detection and treatment. By designing lytic peptides that are activated in the acidic TME, several groups have reported success in treating xenograft models of human cancer. Makovitzki et al., demonstrated the reduction of prostate tumour volumes and angiogenesis using these peptides [267]. Recent studies showed that a technology using the pH low insertion peptide (pHLIP), a peptide that forms α-helix at acidic pH and inserts across cell membranes, could be applied to target tumour cells and image acidic tissue by positron emission tomography in solid tumours in breast and prostate xenografts and in spontaneous murine breast cancer models [268–270]. A recent study suggests that differential uptake of pHLIPs which depend on acidic pHe, may be useful as a predictive biomarker of tumour prognosis [271]. pHLIP-coated liposomes can also be used to insert nanopores caused by the insertion of gramicidin A into the membranes of cancer cells. This hydrophobic peptide forms a β-helix channel that allows protons to enter the cell and decreases intracellular pH, thus inducing apoptosis [272]. Further, because of the lytic action of these peptides, resistance mechanisms are unlikely to develop [267].

Similarly, conditions in the tumour can be utilised to activate pro-drugs that are triggered or become more potent at acidic pH to differentially kill cancer cells. Low pH can increase resistance to some cancer chemotherapeutics by altering charge and structure [13, 25], but pro-drugs have been designed to preferentially target cancer cells and release drugs in the lysosomal or endosomal compartment (pH 5.0). For example, a pro-drug that targets α_v_β_3_ integrin which is overexpressed in certain cancers, and also contains both a fluorescent reporter group and a detachable form of doxorubicin, was recently reported to induce apoptosis *in vitro* in α_v_β_3_ positive glioma cells [273]. Similarly, cytotoxic responses were achieved in cancer cells using pH-responsive silica prodrug nanoparticles to enable the controlled release of doxorubicin in endosomes [274]. This strategy can help overcome intrinsic and acquired multidrug resistance, allowing high intracellular drug concentrations to accumulate. Polyethelyne glycol (PEG) can increase the circulation time of nanoparticles and can be combined with pH-sensitive polymers to allow shedding of PEG in the TME, allowing access of nanoparticles to tumour cells [275]. Cell penetrating peptides such as trans-activator of transcription (TAT) can allow drugs direct access into a cell, but they act in a non-specific manner. This can be overcome using TAT in a smart micelle that shields TAT at normal body pH, but exposes it in the TME if pH falls below 7.0 [276]. Other strategies use the activation of MMPs by the acidic TME to cleave linkers that free cell penetrating peptides only in tumour tissue; these can also be utilised to detect and monitor primary and metastatic tumour growth [277, 278]. The use of pH-sensitive nano-systems including polymers, peptides, as well as inorganic materials, for drug delivery in cancer therapy has been recently reviewed [277, 279]. Phase I clinical trials using cell penetrating peptides have been reported in high grade gliomas and advanced carcinomas [267, 268]. The glioma study used an inhibitor of p53 ubiquitination and reported convincing data showing antitumour activity, with few adverse effects [280]; cell penetrating peptides linked with SN38, the active metabolite of irinotecan, was found to stabilise some advanced carcinomas [281].

The inconsistent nature of the vasculature in tumours means that conditions in the microenvironment can be extremely variable in terms of pH and oxygenation. The ability to measure such changes in real-time would allow treatment to be optimised for maximum efficacy. Radiation treatment is most effective when O_2_ concentrations are high, whereas pro-drugs could be preferentially used when pH is low. At present, O_2_ and metabolic markers can be observed in real-time experiments *in vitro* using microphysiometry methodologies [47]. However, the development of wireless biosensors that can be implanted into solid tumours, to monitor both pH and hypoxia *in vivo* and in real-time is under way; see http://www.see.ed.ac.uk/drupal/impact. This would allow monitoring of tumour conditions to optimise radation and chemotherapy. Such systems have already been used to deliver drugs in an ocular system, and this technology could be further combined with O_2_ and pH sensing [282]. Recently an implantable device was developed that can test up to 16 different drugs/concentration/combinations in tumours *in vivo* [283]. The device was delivered using a biopsy needle, with tumour tissue removed for analysis using a coring needle. A combination of these implantable technologies would greatly improve drug development and would allow the treatment and monitoring of cancer in a truly personalised manner.

## Conclusions

pH is commonly dysregulated in solid tumours, therefore interference with pH regulation is a promising strategy for anti-cancer drug development [13]. Taken together, the acid-base transporters and proton-sensing receptors described above are important for cancer cells to sense and adapt to the acidic tumour microenvironment. Further research is warranted to validate these pH regulators as potential targets for cancer therapy and chemoprevention. As further research into the exploitation of the TME is undertaken, more targets are likely to emerge in this exciting field. Technological advances in drug design and delivery that exploit the acidic nature of solid tumours, in concert with the ability to monitor environmental conditions and treatment responses in real-time, mean that the adaptation of tumour cells to low pH conditions may be exploited in the clinic in the near future.

## Abbreviations

CAIX: carbonic anhydrase IX/9
CyT: cytotoxic T cells
DC: dendritic cells
ECM: extracelular matrix
EGFR: epidermal growth factor receptor
EIPA: ethylisopropylamiloride
GPCR: G-protein coupled receptor
HDAC: histone deacetylases
HIF-1: hypoxia-inducible factor-1
HR: homologous recombination
HUVEC: human umbilical vein endothelial cells
MCT1: monocarboxylate transporter 1
MCT4: monocarboxylate transporter 4
MCTs: monocarboxylate transporters
MDSC: myeloid-derived suppressor cells
MMP: metalloproteases
MDR: multidrug resistant
NHE1: sodium-hydrogen exchanger 1
NK cells: natural killer cells
OXPHOS: oxidative phosphorylation
PEG: polyethelyne glycol
pHe: extracellular pH
pHi: intracellular pH
pHlip: pH low insertion peptide
PPI: protein pump inhibitor
TME: tumour microenvironment
V-ATPase: vacuolar-type H^+^-ATPase proton pump
VEGF: vascular endothelial growth factor

## Declarations

### Copyright

© The Author(s) 2020.
